# Impact of aging-related consumption trend on carbon emission efficiency in China: mediation effect model based on industrial structure adjustment

**DOI:** 10.1007/s11356-023-30400-3

**Published:** 2023-10-19

**Authors:** Ran Yu, Zhangchi Wang, Yan Li

**Affiliations:** School of Environment & Natural Resources, Renmin University of China, Beijing, 100872 China

**Keywords:** Aging-related consumption, Carbon emission efficiency, Industrial structure adjustment, Mediation effect, Regional heterogeneity

## Abstract

With the deepening of China’s aging process, changes in the age structure of the population affect the industrial structure and consumption structure in different ways and have a knock-on effect on the whole economic system. Therefore, aging is one of the objective factors affecting future carbon emissions in China. This study analyzes the impact mechanism of aging-related consumption trend on carbon emission efficiency (CEE) based on panel data of 30 Chinese provinces from 2000 to 2019. The results show that the aging-related consumption trend is conducive to the improvement of regional CEE, and the mediation transmission mechanism of industrial structure adjustment is obvious, with a coefficient of 0.1496. The core industry closely linked to the demand for aging-related consumption is consumer services. The promotion of the consumption demand of the aging in the eastern region on the CEE and the transmission stimulation of the industrial structure adjustment are the most obvious. The mediation effect in the central and western regions is relatively weak, and the aging-related consumption demand has not formed a positive interaction with the aging industry. Therefore, improving the market construction of products and services for the aging is beneficial to achieve a virtuous cycle of aging-related consumption upgrading and carbon emission efficiency. This research can provide insights for China to promote industrial structure transformation within the aging trend and also help China meet its carbon neutrality target on schedule.

## Introduction

Global warming caused by greenhouse gas emissions is considered to be an unconventional security threat, and it has become a consensus that sustainable development must be based on reducing greenhouse gas emissions (Böhringer et al. 2012; Liu et al. 2015). In China, carbon dioxide emission accounts for about 83% of greenhouse gases, and reducing CO_2_ emission is the key to controlling the quality of the atmospheric environment. The intensity of carbon emission is closely related to the characteristics of industrial structure, among which the tertiary industry has the lowest carbon emission intensity (Cheng et al. 2018; Wang et al. 2021). So the adjustment and upgrading of industrial structure and the optimization of factor resource allocation are necessary ways to balance China’s economic development and carbon emission reduction targets (Li and Wang 2019; Zhang et al. 2018a, b, 2023a). The current population age structure in China is facing profound changes and has a chain reaction in the economic system. The aging of the population is causing the “demographic dividend” of economic growth to fade, but meanwhile, the shift in consumption patterns brought by the growth of the elderly population is an opportunity to drive the evolution of an advanced industrial structure. The rise in the proportion of elderly population will drive the growth of service-oriented consumption (Mao and Xu 2014), thus forcing the upgrading of industrial structure. It will determine the process of China’s carbon emission control in the mid- and long-term (Guo et al. 2023). Therefore, it is essential to consider the dynamic impact of aging trend when formulating low-carbon industrial policies.

Population aging is an important objective factor affecting industrial structure adjustment. On the one hand, population aging reduces the number of the working-age population and labor productivity and restricts the adjustment of industrial structure (Chen and Wang 2023; Feyrer 2007). On the other hand, the aging population effectively forces China to upgrade its industrial structure through the accumulation of human capital and the expansion of demand (Guo et al. 2023; Shen et al. 2022; Liu and Peng 2016). Compared to the supply-side factors, the demand-side factors are the endogenous driving force of the aging population to affect the industrial structure. The irreversible physiological and psychological changes caused by human senescence determine the rigidity of the demand for aging products and services (Vrhovec and Tajnikar 2016).

China’s aging population trend has coexisted with rapid economic growth for a long time (Cai 2021). The level of consumption corresponding to population aging in each stage of economic development varies (Sungja et al. 2021). The habitual characteristics and the differences in the living space and social changes experienced by elderly in different birth cohorts lead to differences in the consumption patterns of older populations in each age group (Mao and Xu 2014). At current stage, China’s oldest seniors belong to the pre-1945 birth cohort. They have experienced the war turmoil and national reconstruction, and the lack of material life in their middle and young adulthood, thus forming habits of hardship and frugality (Seoseonyoung et al. 2021). Those born in the 1945–1955 cohort are middle-aged seniors, growing up in a period of tortuous advancement and economic broader reform in China, whose middle and old age have benefited from the dividends of reform and opening up. Their consumption habits are similar to those of the previous period, with basic physiological demands as the guide for consumption (Wang et al. 2022a, b). The elderly born after 1955 belong to the younger seniors, who participated in the wave of reform and opening up during their youth, and experienced the rapid development of Internet technology during their middle age (Wang and Yu 2020). They have accumulated rich assets in their old age and have a modern consumption concept with the sense of pursuing personalized consumption (Sungja et al. 2021).

With the deepening of the aging process, the change of generations makes the consumption demand of the elderly tend to diversify (Kuhn and Prettner 2018). In order to meet the decline of physiological functions in the elderly, the demand for service products increases (Yang et al. 2021). Simultaneously, economic development drives up the spiritual consumption demand (Erlandsen and Nymoen 2008), transforming the social consumption structure. It will directly promote the development of silver-haired industries, such as technology-intensive industries and consumer services (Liu and Peng 2016), and will have a long-term impact on China’s low-carbon economy development. In this context, it is necessary to analyze whether the transformation of consumption demand caused by population aging can force the industrial restructuring and ultimately affect China’s carbon emission efficiency. That is of great importance to achieve the “peak carbon” and “carbon neutral” goals as scheduled.

This study will adopt industrial structure adjustment as the mediation variable to analyze the mechanism of aging-related consumption trend on carbon emission efficiency. The aims and main contribution of this research are to (1) quantify aging-related consumption trend and analyze the mechanism of its impact on regional carbon emission efficiency; (2) verify the mediation effect of industrial structure adjustment and further consider two specific types of aging-related industries to explain the endogenous driving force of aging-related consumption trend on industrial structure adjustment; and (3) investigate the regional heterogeneity of aging-related consumption trend and mediation effects to identify the carbon emission reduction potential in specific regions. The results of this study can provide scientific guidance for China to formulate low-carbon industrial development policies in the context of population aging.

## Literature review

Besides the primary energy structure (Fan et al. 2023), economic scale (Sheldon 2019), foreign trade (Li and Wei 2021), and industrial structure (Sun et al. 2020), the age structure of the population is also an important factor influencing carbon emission intensity (Dalton et al. 2008; Menz and Welsch 2012; Wang and Wang 2021). This section will clarify how the aging-related consumption demand trend affects carbon emission by changing the industrial structure in the context of population aging based on existing literature.

### The transformation of consumption demand structure in the context of aging

Compared with developed countries, China has entered an aging population in an old before getting rich cases. Due to the deep-rooted frugal lifestyle habits of the current generation of elderly, it was widely believed that the aging trend of the population has depressed social consumption expenditure in China (Seoseonyoung et al. 2021; Wang and Yu 2020). But as the quality of China’s economic development continues to improve, this phenomenon has changed (Kuhn and Prettner 2018). The precautionary saving that comes with increased life expectancy may partially hinder the spending power of the elderly (Hu 2015; Wang and Ai 2015). However, as the elderly reach a significantly higher level of education and income, they will have a greater propensity and ability to consume at an older age (Qi and Liu 2020).

The consumption of the elderly is significantly different from that of other age groups. Aging-related consumption demands tend to be health-oriented and hedonistic due to the decline of physical functions and the pursuit of spiritual satisfaction (Tian et al. 2015). The rise in the number of older adults will significantly drive up healthcare consumption expenditure (Yang et al. 2021; Zhang et al. 2017; Zhang et al. 2018a, b), and the consumption behavior of the elderly focus on the comfort and convenience of life (Wang and Li 2021), promoting the expansion of the corresponding industries and services in society (Mao and Xu 2014; Qi and Liu 2020). In addition, changes in the lifestyle of the elderly will also affect consumption. With the elderly spending more time at home, residential consumption rises, leading to increased household energy consumption (Yagita and Iwafune 2021; Zhu and Lin 2022) and more carbon emissions (Yu et al. 2023a, b; Zheng et al. 2022), which is also positively correlated with the age of homebound older adults (O’Neill and Chen 2002).

To summarize, the rise in the proportion of the elderly in the total population will obviously stimulate the growth of the development of enjoyment-oriented consumption and, to a certain extent, drive the household energy consumption.

### Aging-related consumption trend and industrial structure adjustment

Domestic demand is an endogenous driver of the industrial economic growth. Aging-related consumption demand requires society to provide more aging products and supporting services (Lee and Mason 2010; Lu et al. 2018). Due to the physiological characteristics of the elderly, they have a high preference for medical services (Yang et al. 2021). The aging leads to an increase in the demand for health care, which brings a shift in the labor force from other labor production sectors to the health care sector (Hashimoto and Tabata 2010). Beyond the basic health needs, because of the richness of life experience and the growth of demand hierarchy brought by age, aging will also drive the development of household services, senior education, senior tourism, and other consumer services (Wang et al. 2022a, b; Yenilmez and Meltem 2015). And with the deepening of population aging, the aging industry can receive more government industrial policy support (Lee and Mason 2010). It is beneficial to the transition from the secondary to the tertiary sector, thus helping to upgrade the industrial structure.

Aging-related consumption trend is one aspect of the impact of aging on the industrial structure, and its impact on the supply side is reflected in that it changes the labor allocation. Population aging reduces the proportion of younger workers, decreases labor productivity in society (Dostie 2011; Hernæs et al. 2023), and will increase the financial burden (Hock and Weil 2012). The aging affects the allocation of labor resources and thus has a negative impact on labor-intensive industries, but it also has the effect of forcing human capital accumulation and promoting the development of technology-intensive industries (Annabi et al. 2009; Kim and Song Lee 2023).

Nonetheless, compared to labor supply, population aging still has a more direct impact on industrial adjustment on the demand side, which is reflected in the rise of the proportion of the elderly changing the social consumption structure (Erlandsen and Nymoen 2008), leading to a shift in the direction of industrial development (Wakabayashi and Hewings 2007; Chu et al. 2017a, b). Based on this, we propose research hypothesis 1: Aging-related consumption trend will drive the growth of consumer services, thus contributing to the industrial structure upgrading.

### The impact of industrial structure upgrading on carbon emission

The growth of the service sectors driven by aging-related consumption trend is consistent with the direction of industrial structural upgrading. Advanced industrial structure manifests in the proportion of the tertiary sector in the national economy will continue to rise with the increase in the level of economic development. Although the economic activities of all three industries depend on energy consumption (Chunmei et al. 2011), the secondary industry has the highest energy intensity and pollution emissions (Panayotou 1997). The carbon emission intensity of the tertiary industry is significantly lower than that of the secondary industry (Wu et al. 2021; Zhang et al. 2019). Among the tertiary sectors, the energy consumption of consumer services is lower than that of the transportation sector (Fourcroy et al. 2012). Therefore, expanding the scale of tertiary services in the economic structure and upgrading the industrial structure is a crucial way to reduce the total carbon emissions and improve energy use efficiency (Tian et al. 2014).

So we propose research hypothesis 2: Aging-related consumption trends can contribute to the improvement of carbon emission efficiency by driving industrial structure upgrading.

Existing research mainly considered the impact of population aging on production or consumption, or directly explored the influence of aging or industrial structure on carbon emissions. But these studies fail to establish an effective linkage of the mechanism path of “aging-related consumption trend–—industrial structure adjustment—carbon emission efficiency.” There is a lack of quantification of aging-related consumption trends. And there is also a gap in analyzing whether aging-related consumption trend changes carbon emission efficiency by driving adjustment of specific industrial structure. The impact of population aging on industrial structure adjustment is an important intermediate mechanism affecting carbon emission intensity. By driving the transformation of consumption structure, population aging can promote the development of silver-haired industries, which are mainly tertiary sectors. Thus, the increase of aging-related consumption demand theoretically helps to promote the upgrading of industrial structure and then improves the carbon emission efficiency in the process of economic development. This study will conduct an empirical test on this mechanism.

## Methodology

### Super-SBM-DEA model based on undesired output

This study uses the super-slacks-based model data envelopment analysis (Super-SBM-DEA) the SBM model based on undesirable output to measure the carbon emission efficiency of 30 provinces in China (except Hong Kong, Macao, Taiwan, and Tibet) from 2000 to 2019. The SBM model introduces slack and residual variables into the linear programming equation model, compensating for the neglect of the input–output slack problem in traditional data envelopment analysis (DEA) models (Tran et al. 2019). The efficiency values obtained from the SBM model are in the range of [0, 1], but in most situations, there may be multiple efficient decision-making units (DMUs) with efficiency values equal to 1. Therefore, this study uses the Super-SBM-DEA model to estimate carbon emission efficiency, which can have an efficiency evaluation value of 1 or more, and thus can effectively rank the DMUs with efficiency values higher than 1.

Assuming that there are *n* decision units in the carbon efficiency assessment (*j* = 1,2,⋯,*n*), each with *m* input indicators *x*_*i*_ (*i* = 1,2,⋯,*m*), *q*_*1*_ desirable outputs *y*_*r*_ (*r* = 1,2,⋯,*q1*), and q_2_ undesirable outputs *b*_*t*_ = (*t* = 1,2,⋯,*q*_*2*_). The model is as follows:

1 $${\text{min}}\rho =\frac{\frac{1}{m}\sum\limits_{i=1}^{m}\frac{{S}_{i}^{-}}{{x}_{ik}}}{1-\frac{1}{{q}_{1}+{q}_{2}}(\sum\limits_{r=1}^{{q}_{1}}\frac{{S}_{r}^{+}}{{y}_{ik}}+\sum\limits_{t=1}^{{q}_{2}}\frac{{S}_{t}^{b-}}{{b}_{rk}})}$$

2 $$\left\{\begin{array}{l}\sum\limits_{j=1,j\ne k}^{n}{x}_{ij}{\lambda }_{j}-{S}_{i}^{-}\le {x}_{ik}\\ \sum\limits_{j=1,j\ne k}^{n}{y}_{rj}{\lambda }_{j}+{S}_{r}^{+}\ge {y}_{rk}\\ \begin{array}{l}\sum_{j=1,j\ne k}^{n}{b}_{ij}{\lambda }_{j}+{S}_{t}^{b-}\ge {x}_{ik}\\ 1-\frac{1}{{q}_{1}+{q}_{2}}(\sum\limits_{r=1}^{{q}_{1}}\frac{{S}_{r}^{+}}{{y}_{ik}}+\sum\limits_{t=1}^{{q}_{2}}\frac{{S}_{t}^{b-}}{{b}_{rk}})>0\end{array}\end{array}\right.$$

In Eq. (2),* ρ* is the carbon efficiency of the DMU.${S}_{i}^{-}$.${S}_{r}^{+}$.${S}_{t}^{b-}$ are slack variables for the input indicator, desirable output, and undesirable output, respectively; *λ* is the weight. A DMU’s efficiency value for the year is efficient if the efficiency value* ρ* ≥ 1.

In an undesirable output-based SBM model, each decision unit includes several input variables and output variables. In this study, the input variables are labor force, fixed capital, and energy consumption. The desirable output is the gross regional product, and the undesirable output is the CO_2_ emission. The description of input and output variables is shown in Table 1.

**Table 1:** Input–output variables for the Super-SBM-DEA model with undesirable output

Variable type	Variable name	Data description
Inputs	Labor force	Total employment in the three sectors by region per year
	Capital	This study uses the perpetual inventory method to estimate the fixed capital stock
	Energy	Annual energy consumption by region
Desirable output	Economic output	Real GDP by region, deflated using nominal GDP and GDP indices for each province (adjusted to 2000 base period) to obtain real values
Undesirable output	CO_2_ emission	Estimated according to the methodology provided in the IPCC guidelines

CO_2_ emission from energy consumption (except heat and electricity) is estimated using the methodology recommended by the IPCC:

3 $$C{O}_{2 j}={E}_{j}\times {G}_{j}\times {A}_{j}\times {B}_{j}\times \frac{44}{12}$$

In Eq. (3), CO_2*j*_ is the CO_2_ emission of the *j* energy; *E*_*j*_ is the consumption of the *j* energy (including raw coal, coke, gasoline, diesel oil, kerosene, fuel oil, and natural gas); *G*_*j*_ is the net calorific value of the *j* energy; *A*_*j*_ is the CO_2_ emission coefficient; and *B*_*j*_ is the carbon oxidation factor.^1^

Electricity and heat are different from their traditional fossil counterparts. The annual carbon emission factors for electricity and heat depend on the type and proportion of energy consumed in the energy conversion process. In this paper, the carbon emission factors for electricity and heat are calculated using the following equation:

4 $$CO{E}_{h}^{t}=\frac{\sum C{H}_{i}^{t}}{{\text{Heat}}^{t}}$$

5 $$CO{E}_{e}^{t}=\frac{\sum C{E}_{i}^{t}}{El{e}^{t}}$$

Among them, ${COE}_{h}^{t}$ and ${COE}_{e}^{t}$ are the carbon emission coefficients of heat and electricity in year *t* respectively; ${CH}_{i}^{t}$ and ${CE}_{i}^{t}$ are the carbon emissions generated by the *i* energy consumed of heat and electricity production in year t respectively; ${\mathrm{Heat}}^{t}$ and ${Ele}^{t}$ are the total consumption of heat and electricity in year *t* in China.

### Mediation effects model

This paper uses a mediation effects model to quantify the impact of the rising demand for aging-related consumption on carbon emission efficiency. First, the Tobit model is adopted as a benchmark model to analyze the impact of population aging and consumption structure on carbon emissions efficiency. The Tobit model is a limited dependent variable regression model that describes the association between a non-negative dependent variable and the independent variables when data are censored or truncated. Since the relative efficiency scores obtained from the Super-SBM-DEA model are a series of discrete variables that are censored from the left-hand side of 0 (Li and Zeng 2020). The Tobit model can effectively avoid the biased parameter estimation of the ordinary least squares method due to the truncation of the efficiency score and can mitigate the interference of heteroskedasticity (Li and Zeng 2020). So in this study, the Tobit model is chosen to analyze the factors influencing carbon emission efficiency, and the form is as follows:

6 $$CEE={\alpha }_{0}+{\alpha }_{1}\mathrm{age}+{\alpha }_{2}\mathrm{cons}+{\alpha }_{3}\mathrm{age}*\mathrm{cons}+\beta X+\varepsilon$$

where *CEE* is carbon emission efficiency; *age* represents the dependency ratio of the elderly population: and *cons* is the consumption structure. Since population aging promotes the upgrading of consumption structure related to the demand of the elderly, there is a synergistic effect between aging and consumption structure when considering its effect on carbon emission efficiency, so this study constructs an interaction term of age*cons and uses it to reflect the aging-related consumption trend. *X* represents a series of control variables, including regional economic development level (*pgdp*), foreign direct investment (*fdi*), technological innovation (*tech*), energy structure (*es*), urbanization level (*urz*), and human capital (*hc*).

Logically, the aging-related consumption trend should affect the carbon emission efficiency through the transmission mechanism of adjusting the industrial structure. In order to identify whether this transmission mechanism exists, this study constructs the following mediation effect model:

7 $$CE{E}_{it}={\beta }_{0}+{\beta }_{1}{\mathrm{age}}_{it}+{\beta }_{2}{\mathrm{cons}}_{it}+{\beta }_{3}{\mathrm{age}}_{it}*{\mathrm{cons}}_{it}+\omega {X}_{it}+{\varepsilon }_{it}$$

8 $${M}_{it}={\lambda }_{0}+{\lambda }_{1}{\mathrm{age}}_{it}+{\lambda }_{2}{\mathrm{cons}}_{it}+{\lambda }_{3}{\mathrm{age}}_{it}*{\mathrm{cons}}_{it}+\gamma {X}_{it}+{\varepsilon }_{it}$$

9 $$CE{E}_{it}={\theta }_{0}+{\theta }_{1}{\text{age}}_{it}+{\theta }_{2}{\text{cons}}_{it}+{\theta }_{3}{\text{age}}_{it}*{\text{cons}}_{it}+\eta {M}_{it}+\delta {X}_{it}+{\varepsilon }_{it}$$

where *M*_*it*_ is the mediation variable, including the industrial structure adjustment (*ind*), the proportion of high-tech industries (*ahti*), and the per capita output value of the health and resident services industry of the elderly population (*hrs*). When the estimated results of *λ*_*3*_ and *η* are significant, the mediation effect is valid. If at least one of* λ*_*3*_ and *η is* not significant, the existence of the mediation effect needs to be determined by Sobel’s test.

## Variable description

### Explained variables

#### Carbon emission efficiency (CEE)

**Fig. 1:**
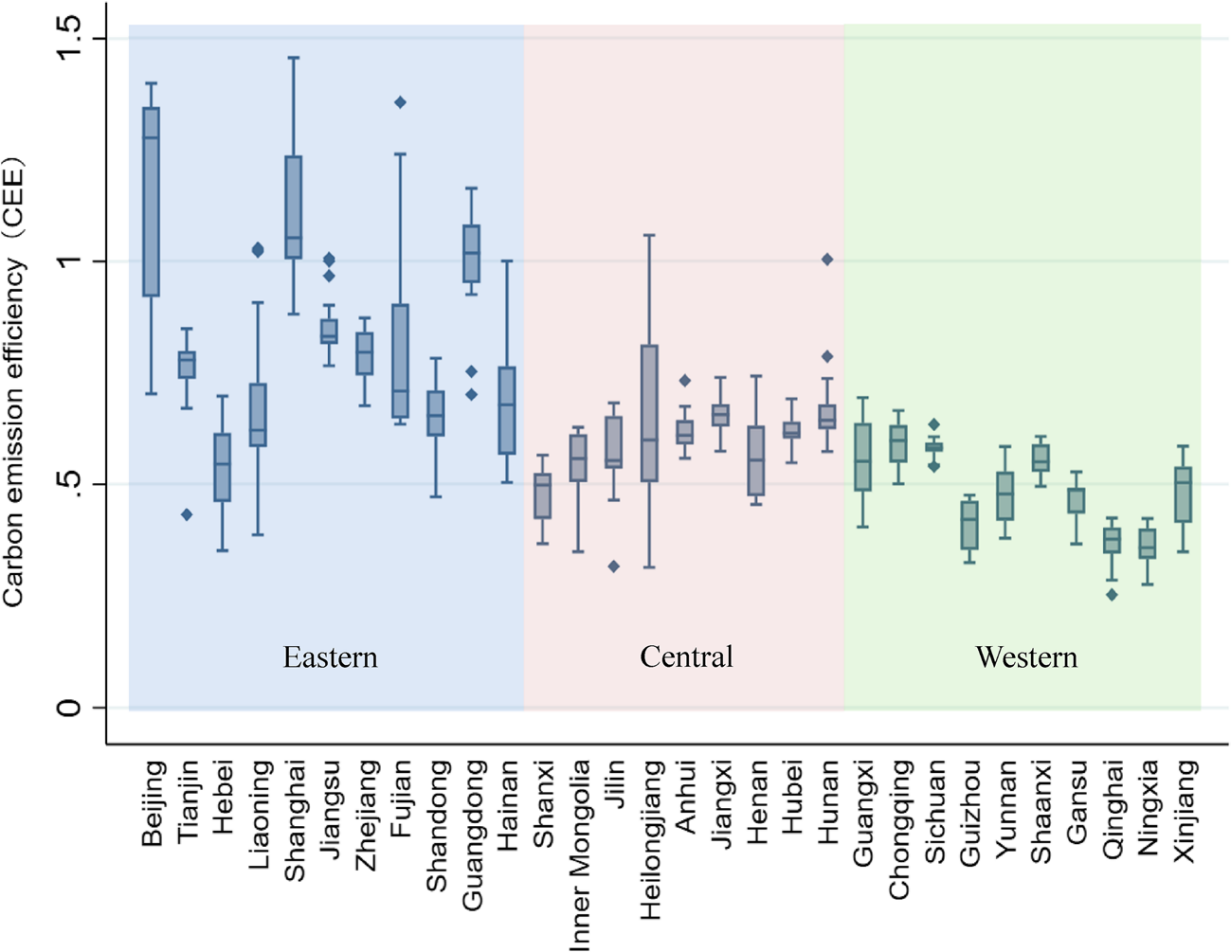
Box line plot of carbon emissions efficiency for 30 Chinese provinces, 2000–2019

The calculation results of the Super-SBM-DEA model based on undesirable output are shown in Fig. 1. The provinces with the highest CEE are Beijing, Shanghai, and Guangdong, corresponding to the three most economically developed regions in China. For the regional heterogeneity, the CEE of provinces in the eastern region is significantly higher than those in the central and western regions. The CEE of the western region is generally lower than the national average. It confirms the existence of a certain positive correlation between carbon emission efficiency and the level of regional economic development. Hebei, Shanxi, and Henan are heavy industrial regions with a high proportion of secondary industries, and their economic development is overly dependent on local natural resource exploitation and energy consumption, resulting in the CEE remaining at a low level.

### Core explanatory variables

#### Population aging (*age*)

The elderly dependency ratio is chosen to measure the degree of aging in a region, expressed as the share of the population aged 65 and over in the working age (15–64) population (Lu et al. 2018).

#### Consumption structure (*cons*)

This paper aims to measure the structure of aging-related consumption. The rise in the elderly will help to promote the development of enjoyment-based consumption to increase (Mao and Xu 2014; Qi and Liu 2020). In addition, as the elderly spend more time at home, household energy consumption will also rise (Yagita and Iwafune 2021). Therefore, this paper selects four types of consumption, namely health care expenditure, cultural, educational and recreational consumption, household equipment supplies and services consumption, and housing consumption, as consumption expenditure is closely related to the elderly population, and calculates their proportion of the overall expenditure to characterize the consumption structure.

#### Aging-related consumption trend (age*cons)

**Fig. 2:**
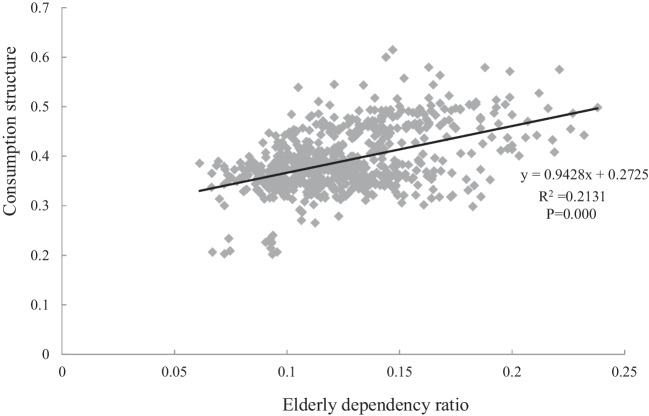
Correlation analysis between population aging and consumption structure

This study characterizes the aging-related consumption trend by constructing an interaction term between population aging (*age*) and consumption structure (*cons*). As shown in Fig. 2, the correlation analysis of the 30 provinces shows that the elderly dependency ratio is significant positively correlated with the consumption structure. It means that there is a synergistic effects between population aging and consumption structure, so a rise in the value of age*cons represents an upgrading of aging-related consumption demand, indicating a deepening of aging-related consumption trend.

### Mediation variables

#### Industrial structure adjustment (*ind*)

The supply-side transmission mechanism of the aging-related consumption trend is mainly reflected in the expansion of the tertiary services sector (Yu et al. 2023a, b). This study uses the industrial structure upgrading index, i.e., the ratio of the tertiary sector to the secondary sector, to indicate the industrial structure adjustment.

#### The proportion of high-tech industries (*ahti*)

The rise in the proportion of technology-intensive industries is the main feature of the upgrading of the low-carbon structure within the secondary industry (Zhang et al. 2023a, b). Thus, the ratio of the main operating revenue of pharmaceutical and medical equipment manufacturing to the overall manufacturing output value is used to measure the proportion of aging-related high-tech industries according to the physiological demand characteristics of the elderly.

#### Per capita silver-haired services output for the elderly (*hrs*)

Two major service industries closely related to the aging demands, the health and residential service sectors (Sungja et al. 2021), are chosen to measure the silver-haired services. Due to data limitation, we use the product of the number of urban employees in these sectors and the output value per person employed in the tertiary sector to approximate the annual output value of the silver-haired services.

### Control variables

#### Gross regional product per capita (pgdp)

The actual GDP of each region is divided by the total population. Per capita GDP reflects the level of economic development of a region, which is closely related to carbon emission efficiency (Zheng et al. 2020a, b).

#### Foreign direct investment (fdi)

The total import and export of goods by foreign-invested enterprises is selected as a proxy variable for FDI. According to the “pollution halo” (Tian et al. 2023) and “pollution paradise” (Jiang et al. 2022) hypotheses, there are two-sided effects of FDI on local environmental quality.

#### Technological innovation (tech)

Measured by the number of annual patent applications granted locally. Technological innovation is a direct and effective way to reduce carbon emissions per unit of output (Jin et al. 2022).

#### Energy structure (cs)

The proportion of coal consumption is used to measure the energy structure.

#### Urbanization rate (urz)

This is measured using the proportion of urban population in the total population. An increasing level of urbanization is closely linked to the transformation of the local industrial structure (Yao et al. 2023).

#### Human capital (hc)

This is measured by the years of education per capita in a region. Human capital is necessary to drive technological progress (Wu and Liu 2021), and its role in the restructuring of local industries and the improvement of carbon intensity cannot be ignored (Yu et al. 2023a, b).

The raw data for all variables are obtained from China Statistical Yearbook, China Regional Economic Statistics Yearbook, China Energy Statistical Yearbook, China Labor Statistics Yearbook, and the statistical yearbooks of each province published in previous years. The descriptive analysis of the main variables is shown in Table 2.

**Table 2:** Descriptive statistics

Variable	Definition	Unit	Obs	Mean	Std. dev	Min	Max
CEE	Carbon emission efficiency	/	600	0.643	0.221	0.253	1.456
age	Elderly dependency ratio	/	600	0.128	0.031	0.061	0.238
cons	Consumption structure	/	600	0.396	0.059	0.266	0.615
ind	Industrial structure upgrading index	/	600	1.014	0.541	0.494	5.169
ahti	The proportion of high-tech industries	%	600	3.125	1.799	0.257	11.89
hrs	Per capita silver-haired services output for the elderly	Thousand yuan/person	600	6.271	5.853	0.552	43.63
pgdp	Gross regional product per capita	Thousand yuan/person	600	9.936	5.362	2.645	29.66
fdi	Foreign direct investment	Billion dollar	600	43.53	96.99	0.004	592.1
tech	Technological innovation	Thousand	600	28.02	56.78	0.07	527.4
cs	The proportion of coal consumption	%	600	21.01	10.22	0.588	82.94
urz	Urbanization level	%	600	51.18	15.19	23.3	89.6
hc	Human capital	Year	600	9.198	1.322	6.089	13.91

## Results and discussion

### Benchmark results

As shown in Table 3, the coefficient of *age* is significantly negative, indicating that the process of population aging reduces carbon emission efficiency, which is closely related to the characteristics of the scale of labor-intensive industries in China. However, the coefficient of the interaction term age*cons are significantly positive, and the value in the Tobit model (model (2) in Table 3) is 7.4123, indicating that the upgrading of the aging-related consumption demand is a key factor in improving the efficiency of carbon emission. The net effect of *cons* on carbon efficiency is negative. It is related to that the consumption structure measured in this paper includes consumption of household equipment and daily necessities and housing energy consumption. When considering the impact of consumption structure on carbon efficiency, the scale of residents’ consumption of industrial manufacturing products and household energy is larger than that of products in the tertiary sector.

**Table 3:** Baseline regression results

	Tobit model		Fixed effects model	
	(1)	(2)	(3)	(4)
age	− 2.2339^*^	− 3.5243^***^	− 2.4662^**^	− 3.6287^***^
	(1.1466)	(1.0433)	(1.1737)	(1.1020)
cons	− 1.0784^**^	− 1.5623^***^	− 1.1751^***^	− 1.5826^***^
	(0.4312)	(0.3955)	(0.4410)	(0.4137)
age*cons	5.4835^**^	7.4123^***^	5.7425^**^	7.5862^***^
	(2.5933)	(2.3783)	(2.6487)	(2.4733)
pgdp		0.2915^***^		0.3550^***^
		(0.0472)		(0.0509)
fdi		0.0131^**^		0.0176^***^
		(0.0058)		(0.0068)
tech		0.0464^***^		0.0640^***^
		(0.0111)		(0.0152)
urz		− 0.4259^**^		− 0.6617^***^
		(0.1660)		(0.2055)
cs		− 0.2726^***^		− 0.2837^***^
		(0.0639)		(0.0671)
hc		0.5163^***^		0.5831^***^
		(0.1157)		(0.1298)
_cons	1.1367^***^	− 2.5290^***^	1.1861^***^	− 3.3026^***^
	(0.1802)	(0.4055)	(0.1809)	(0.4665)
Provincial fixed effect			Yes	Yes
Time fixed effect			Yes	Yes
*N*	600	600	600	600
*R*^2^			0.2538	0.4186

Note: ^***^, ^**^, and ^*^ indicate significance at the 1%, 5%, and 10% levels

Aging leads to a rise in the average age of the labor force, which is an important factor affecting the physical state and skill level of workers (Feyrer 2007). The older labor force will cause a decrease in unit labor productivity. In addition, aging leads to an increase in the difficulty and cost of learning new knowledge for workers (Börsch-Supan and Weiss 2016), making it difficult for them to adapt to the development requirements of high-technology sectors. The average quality of the workforce decreases, which is not conducive to productivity.

But on the demand side, aging-related consumption trend is directly related to the increase of the income level and consumption ability of the elderly (Addessi 2018). With the upgrading of the consumption structure, the market demand for aging products and services expands, which leads to the adjustment of industries (Hock and Weil 2012). The industrial sectors closely related to the demand of the elderly are mainly tertiary industries, whose energy consumption per unit of product production is lower compared to other industries, thus contributing to the improvement of carbon emission efficiency.

According to model (2) in Table 3, the coefficients of *pgdp*, *fdi*, *tech*, and *hc* are significantly positive, implying that each of these control variables has a positive effect on the regional carbon emission efficiency improvement.

Regional economic development is conducive to the improvement of carbon emission efficiency. This is due to China’s transition from a “crude” economic growth model to an “intensive” sustainable development model (Gu et al. 2021; Yang et al. 2022), which has enabled the development of developed regions to gradually break away from their dependence on energy inputs (Zheng et al. 2020a, b). As incomes rise, residents have the will and ability to consume products from the service sector, thus promoting the development of the tertiary sector.

The purpose of technological innovation activities by enterprises is to reduce marginal production costs, which are exogenously motivated by government policies on environmental regulation (Song et al. 2019). Thus enterprises’ R&D investments in science and technology are focused on improving cleaner production efficiency (Chen et al. 2022), bringing a reduction in the intensity of energy consumption in industrial economic activities.

Human capital accumulation can significantly promote a low-carbon economy. The reason is that highly educated workers have comprehensive and integrated skills (Sánchez-Romero et al. 2016), thus facilitating the development of technology and knowledge-intensive industries (Wu and Liu 2021).

This study also supports the “pollution halo” hypothesis, suggesting that foreign investment has a technology spillover effect (Tian et al. 2023). By opening up the market, foreign investment can bring advanced green production technologies to local enterprises (Wang and Luo 2020), thus leading to cleaner production efficiency in the local industry.

### Mediation effects of industrial structure adjustment

The benchmark results verify that aging affects carbon efficiency on both the supply and demand sides. We further incorporate aging-induced industrial structure upgrading as a mediation variable in the analysis. As shown in Table 4, the coefficient of *age* is significantly negative (model (2) in Table 4), while the coefficient of age*cons is significantly positive, indicating that the upgrading of the consumption demand due to population aging is an important way for it to promote the advanced adjustment of industrial structure. After controlling for the demand-side effects, the supply-side factors such as the rise in the average age of the workforce and the decline in labor productivity will hinder the upgrading of the industrial structure (Chen and Wang 2023; Feyrer 2007).

**Table 4:** Tests of the transmission mechanism of industrial restructuring as a mediation variable

	Using ind as the explanatory variable		Using CEE as the explanatory variable	
	(1)	(2)	(3)	(4)
age	− 12.1134^***^	− 5.7098^**^	− 0.8510	− 2.1771^**^
	(2.5524)	(2.3195)	(1.1398)	(0.9755)
cons	− 0.5260	0.2314	− 1.0000^**^	− 1.5909^***^
	(0.9595)	(0.8702)	(0.4205)	(0.3650)
age*cons	21.7280^***^	11.4614^**^	3.0363	4.8387^**^
	(5.7764)	(5.2083)	(2.5598)	(2.2065)
ind			0.1103^***^	0.1496^***^
			(0.0179)	(0.0169)
pgdp		− 0.3813^***^		0.3319^***^
		(0.1068)		(0.0507)
fdi		0.0061		0.0118^**^
		(0.0141)		(0.0055)
tech		0.0673^**^		0.0471^***^
		(0.0312)		(0.0106)
urz		− 3.7561^***^		− 0.2595^*^
		(0.4339)		(0.1574)
cs		− 0.1318		− 0.2639^***^
		(0.1412)		(0.0590)
hc		0.4268		0.3798^***^
		(0.2728)		(0.1118)
_cons	1.4855^***^	4.4308^***^	0.9626^***^	− 2.8248^***^
	(0.4011)	(0.9906)	(0.1772)	(0.4835)
*N*	600	600	600	600

Note: ^***^, ^**^, and ^*^ indicate significance at the 1%, 5%, and 10% levels

As shown in model (4) in Table 4, the coefficient of the core explanatory variables did not change significantly after the addition of the mediation variable. The coefficient of the mediation variable (*ind*) is significantly positive, indicating that there is a significant mediation effect. The expansion of the scale of the tertiary sector contributes to the improvement of carbon emission efficiency in production by gradually replacing the excess capacity of the secondary sector. Therefore, the research hypothesis 2 is proved to be correct.

With the addition of the mediation variable, the value of age*cons decreases from 7.4123 to 4.8387, proving that the aging-related consumption trend is contributing to the improvement of the regional carbon emission efficiency level by promoting the advanced industrial structure. In the context of an aging population, the growing demand for aging-related consumption will lead to a rapid increase in products and services in the silver-haired sectors due to the transmission mechanism between the supply and demand structure (Shen et al. 2022; Wang and Yu 2020). Most of the silver-haired industries are oriented towards technological and professional skill inputs and are tertiary industries (Xu and Liu 2023). This means that regional economic growth is much less dependent on energy inputs, which is conducive to reducing energy consumption per unit of output and correspondingly improving the regional carbon emission efficiency.

### Robustness tests

To test the robustness of the estimation results of the baseline regression, this section chooses to replace the measure of population aging by using the ratio of the population aged 65 years or older to the total population at the end of the year in each province. The results are shown in Table 5. The estimated results and significance of the main explanatory variables remain largely consistent with those in Tables 3 and 4, where *age* still has a significant negative effect on *CEE*, and the coefficient of age*cons remaining significantly positive. In the model (3), the effect of *ind* on *CEE* is still significantly positive, which are consistent with the previous results, indicating that aging-related consumption trend does contribute to the improvement of carbon emission efficiency by promoting the upgrading of industrial structure. Therefore, the robustness test confirms that the results of the baseline regression and mediation effects model are reliable.

**Table 5:** Robustness tests

	Baseline regress	Mediation effects model	
	(1)	(2)	(3)
age	− 5.7382^***^	− 11.2055^***^	− 3.0987^**^
	(1.4289)	(3.2135)	(1.3592)
cons	− 1.7632^***^	− 0.6039	− 1.5631^***^
	(0.3998)	(0.8833)	(0.3718)
age*cons	11.7420^***^	23.0742^***^	6.2310^**^
	(3.2358)	(7.1659)	(3.0524)
ind			0.1475^***^
			(0.0171)
pgdp	0.2943^***^	− 0.3682^***^	0.3360^***^
	(0.0472)	(0.1064)	(0.0509)
fdi	0.0119^**^	0.0013	0.0119^**^
	(0.0058)	(0.0142)	(0.0055)
tech	0.0486^***^	0.0725^**^	0.0475^***^
	(0.0111)	(0.0311)	(0.0106)
urz	− 0.3706^**^	− 3.5877^***^	− 0.2335
	(0.1655)	(0.4371)	(0.1600)
cs	− 0.2624^***^	− 0.1038	− 0.2604^***^
	(0.0638)	(0.1410)	(0.0590)
hc	0.5073^***^	0.4126	0.3769^***^
	(0.1150)	(0.2715)	(0.1118)
_cons	− 2.4685^***^	4.6338^***^	− 2.8620^***^
	(0.4082)	(0.9856)	(0.4872)
*N*	600	600	600

Note: ^***^, ^**^, and ^*^ indicate significance at the 1%, 5% and 10% levels

### Refined analysis of industrial structure adjustment based on specific aging-related industries

Considering the demand characteristics of the elderly, the scope of the silver-haired industry does not include all tertiary sectors, but rather focuses on the consumer services sector (Sungja et al. 2021), while also involving some technology-intensive manufacturing. Therefore, it is necessary to refine the discussion of the impact of population aging and the change in consumption structure on the development of these specific industries, and examine whether they have a mediation transmission mechanism of aging-related consumption trend affecting carbon emission efficiency. The results are shown in Table 6.

**Table 6:** Tests of transmission mechanisms for high-technology industries and consumer services as mediation variables

	Using ahti as the mediation variable		Using hrs as the mediation variable	
	(1)	(2)	(3)	(4)
age	− 0.0155	− 3.2117^***^	− 15.0353^***^	− 2.2810^***^
	(0.1073)	(1.0201)	(2.7336)	(0.7688)
cons	0.0583	− 1.5140^***^	− 4.4570^***^	− 1.0701^***^
	(0.0411)	(0.3925)	(1.1865)	(0.2769)
age*cons	0.1420	6.6400^***^	25.2659^***^	6.0412^***^
	(0.2420)	(2.3286)	(5.7796)	(1.5756)
ahti		1.7454^***^		
		(0.3800)		
hrs				0.0514^***^
				(0.0196)
Control variables	Yes	Yes	Yes	Yes
*N*	570	570	450	450

Note:^***^, ^**^, and ^*^ indicate significance at the 1%, 5% and 10% levels

First, the proportion of age-related high-tech industries (*ahti*) was used as a mediation variable to analyze the transmission mechanism between the aging-related consumption trend and carbon emission efficiency within the secondary sector. As shown in models (1) and (2) in Table 6, although *ahti* is positively correlated with *CEE*, the effect of age*cons on *ahti* is insignificant, indicating that the deepening of aging does not significantly promote the development of high-technology industries. For Sobel test, the *Z* value is 1.3480 and *P* = 0.1777, meaning the mediation effect is not significant.

Aging-related consumption trend did not significantly stimulate the growth of the medical-related high-tech industry. This is related to the fact that China’s medical high-tech industry is still dominated by national policies and government investment (Chu et al. 2017a, b), and the market-oriented construction of related industries is still imperfect (Wang et al. 2015). The supply of pharmaceutical and medical products and services for specific elderly groups is relatively scarce, resulting in the inability to form effective feedback on the specific needs of the elderly.

Secondly, in order to verify the specific transmission mechanism of the aging-related consumption trend at the tertiary industry level, the per capita silver-haired services output for the elderly (*hrs*) is used as the mediation variable. The results of the empirical analysis are shown in models (3) and (4) in Table 6. When *hrs* is used as the explanatory variable, the coefficient of age*cons is significantly positive, and *hrs* is significantly positively correlated with *CEE*, confirming that the mediation effect stands. The growth of the silver-haired service sectors helps to accelerate the low-carbon development of the industry.

The upgrading of the consumption structure due to population aging in the tertiary sector significantly contributes to the expansion of the output value of health and residential services and other services related to the demand of the elderly. With the deepening of China’s aging population, targeted silver-haired services are developing rapidly to meet the growing material and health needs of the elderly (Seoseonyoung et al. 2021), while positively influencing the upgrading of the industrial structure. It also reflects the initial effectiveness of the development of China’s silver hair industry (Sungja et al. 2021), which can meet the service needs of the elderly to a certain extent.

In model (4) in Table 6, the coefficient of age*cons is significantly positive, with a decrease in absolute value compared to the baseline model (6.0412 < 7.4123), indicating that the growth of the healthcare sector and residential services, which are most closely linked to the elderly in the context of population aging, is the core transmission mechanism through which aging-related consumption trend contributes to the improvement of regional carbon efficiency. It can be concluded that the positive impact of aging-related consumption trend on industrial structure upgrading is mainly reflected in the growth and expansion of the health care and residential services (Liu and Peng 2016). The above results verify that research hypothesis 1 is valid.

### Regional heterogeneity in aging-related consumption trend and mediation effects

Due to the imbalance in China’s regional economic development, there are significant disparities among the development levels of the three industries in different regions. There may be heterogeneity in the socio-economic impacts of population aging in regions with different levels of development. Therefore, this paper classifies the research sample into three sub-samples: eastern, central, and western region^2^ for discussion.

As shown in models (1) and (2) in Table 7, the results of the analysis of the core variables in the eastern region are basically consistent with the full sample. The coefficients of age*cons and *ind* are significant positive. There is a strong mediation effect of industrial structure adjustment. Models (3) and (4) in Table 7 show that the effects of age*cons on *ind*, as well as *CEE* in the central region, are not significant. Whether the mediation mechanism of *ind* holds needs to be determined using the Sobel test. The *Z* value of the Sobel test is 0.3883 and *P* = 0.6978, which cannot reject the original hypothesis, so the mediation effect is not significant. The aging-related consumption trend in the western region has a positive impact on industrial structure upgrading (see in model (5) in Table 7), and the mediation transmission effect is stands (see in model (6) in Table 7).

**Table 7:** Discussion of sub-regional heterogeneity

	Eastern Region		Central Region		Western Region	
	(1)	(2)	(3)	(4)	(5)	(6)
age	− 7.8711^**^	− 3.9966^**^	3.8474	1.4916	− 5.8012^**^	0.3640
	(3.9833)	(1.8308)	(4.5138)	(1.7556)	(2.2797)	(0.7570)
cons	0.5127	− 3.2130^***^	0.4664	1.1287^*^	− 2.0725^***^	− 0.6963^***^
	(1.2769)	(0.7089)	(1.5180)	(0.5848)	(0.7318)	(0.2339)
age*cons	18.1276^**^	9.9513^**^	5.1213	− 5.8444	24.8801^***^	1.8404
	(8.6797)	(3.9022)	(10.5458)	(3.9996)	(5.2777)	(1.7455)
ind		0.3100^***^		− 0.1218^***^		0.0787^***^
		(0.0392)		(0.0242)		(0.0224)
Control variables	Yes	Yes	Yes	Yes	Yes	Yes
*N*	220	220	180	180	200	200

Note:^***^, ^**^, and ^*^ indicate significance at the 1%, 5%, and 10% levels

For the eastern region, aging-related consumption trend can help promote the transformation of the local secondary industry to the tertiary industry, achieve the upgrading of the industrial structure, and, thus, significantly improve local carbon emission efficiency. It is closely related to the high standard of living in the eastern region with a well-developed social welfare guarantee system (Zhang 2017). Above the basic health needs, the elderly has the ability and conditions to pursue cultural and entertainment consumption on a spiritual level (Wang and Li 2021). Due to the solid economic base in the eastern region, the construction of the silver-haired industry is ahead of the country (Sungja et al. 2021), which can effectively support the aging-related consumption demand. Therefore, the consumption demand of the elderly can actually be translated into consumption behavior, driving the scale growth of related industries and promoting the low-carbon development of the economy.

There is no mediation effect of aging-related consumption trend and industrial structure adjustment in the central region. The main reason is related to the fact that the current dominant industries in the central region are still dominated by industrial raw material processing and equipment manufacturing (Zheng et al. 2020a, b). In comparison, the silver-haired industry has not yet formed a certain market scale (Xu and Liu 2023). Due to the supply of products and services lagging behind the demand of the elderly, consumption demands of the elderly beyond health needs are hardly fulfilled (Sungja et al. 2021). The constraints on both the supply and demand sides make the transformation of aging-related consumption trend in the central region lack a positive interaction with sustainable economic development.

For the western region, although aging-related consumption trend has contributed to the upgrading of the industrial structure, the mediation transmission effect on the improvement of carbon emission efficiency is weak. The possible reason is the time period of this study coincides with that of the implementation of the Western Development Plan, and the results of the upgrading of the local industrial structure are more influenced by these national policies. The western region is rich in natural resources and has an important strategic ecological position (Dai et al. 2022); thus, the scale of secondary industry in the western region is less than in the central region (Zheng et al. 2020a, b). And relying on a series of policy support and geographical advantages and reserves of scientific and educational resources, the aerospace, equipment manufacturing, and high-tech industries in the west have grown rapidly and become an important part of the tertiary industry (Wang et al. 2019). As for the elderly itself, the plight of the western and central regions is similar, that is the imbalance between the supply and demand of aging products (Xu and Liu 2023), and therefore the lack of diversity in aging consumer demand, which translates into very limited contribution to the development of a low-carbon economy.

In addition, the impact coefficient of *ind* in the eastern region is significantly higher than that of the central and western regions, reflecting that the advanced level of industry in the developed eastern region is substantially ahead of other less developed regions (Wu and Liu 2021). Environmental regulations in China have become increasingly rigorous in recent years, but there are obvious differences among regions (Mi et al. 2018). The environmental regulations in economically developed regions are more stringent (Peng 2020). The market access threshold for highly polluting and energy-consuming industries has been raised, making it impossible for new highly polluting enterprises to settle in the region (Lian et al. 2016). Meanwhile, developed regions are more able to attract high-level advanced talents (Long et al. 2022), forming a cluster of high-tech industries and tertiary services. Coupled with strong support for these industries in the eastern region, the secondary and tertiary industrial structure in the east is more inclined to cleanliness and technology (Zhang et al. 2022), and the marginal contribution of industrial restructuring is relatively greater.

The impact of *ind* in the central region on *CEE* is negative, and the absolute value of *ind* in the western region is smaller. On the one hand, the central and western regions are rich in energy endowments (Wang et al. 2022a, b), such as natural gas and coal. Resource curse makes the economic growth of the central and western regions depend on energy input (Yang and Song 2019), which limits the development of local high-tech industries. On the other hand, the production technology advantage of the central and western regions is lacking (Chen et al. 2010). Due to the characteristics of resource distribution, local governments prefer to introduce resource-intensive industries (Wang and Chen 2020), resulting in the advanced upgrading of local industries being hindered, which is not conducive to the low-carbon transformation of economic growth.

## Conclusion and implications

### Conclusion

Based on panel data from 30 Chinese provinces from 2000 to 2019, this study empirically investigates the impact of aging-related consumption trend on regional carbon emission efficiency and tests the transmission mechanism of industrial structure adjustment in this context. The main findings are as follows:

(1) the impact of aging-related consumption trend is conducive to promoting the carbon emission efficiency. (2) There is a mediation effect mechanism of industrial structure adjustment. The transformation of China’s aging-related consumption demand has significantly contributed to the adjustment of the advanced industrial structure, thus improving carbon emission efficiency. (3) The core industry closely linked to the demand for aging-related consumption is consumer services, which are mainly health and medical care and residential services. (4) The mediation effect of the industrial structure adjustment on the improvement of carbon emission efficiency in the eastern region is obvious, but that in the central and western regions is relatively weak. The aging demand in the central and western regions fails to form a positive interaction with the low-carbon development of the local economy.

### Policy implications

The upgrading of consumption structure due to aging-related consumption trend can contribute to low-carbon economic growth by promoting the advanced industrial structure and improving the efficiency of regional carbon emissions. As China’s aging process continues to deepen, the government needs to ensure investment in the aging careers and build a sound system of social welfare guarantee to maintain the financial resources of the retired elderly. Chinese government should learn from the multi-level pension system models of developed countries, improving the legally mandatory public pension scheme to provide fundamental guarantee for the elderly in their twilight years. At the same time, enterprises should be urged to pay occupational pensions for their employees, and preferential policies should be introduced to encourage individuals to participate in personal pension savings plans. By broadening individual sources of income in their twilight years, elderly populations can be assured of a consumption capacity that matches their multiple demands.

On the supply side, it is necessary to continuously promote the construction of markets for the silver-haired industries and increase capital investment in corresponding industries such as healthcare and residential services, elderly service facilities, elderly living goods, elderly real estate, elderly tourism, and elderly education, and these industries are clean tertiary industries. The development of these industries will not only stimulate the consumption desire of the elderly but also enhance the level of industrial structure and contribute to sustainable economic development. However, the supply of aging services solely relying on the state’s financial resources is not sufficient, the development of aging-related industries requires diversified sources of funding, and the involvement of social capital can effectively fill the gap. By enriching the investment corpus, the competitive behavior of the market can be utilized to make the allocation of resources in the aging-related service sectors more reasonable.

More importantly, the aging health and service industries require knowledge in a variety of fields, such as medicine, pharmacy, nursing, and psychology. There is a need to accelerate the training of talents and promote the specialization of practitioners in aging-related industries. Government departments can cultivate a group of high-level integrated talents for aging health services by cooperating with universities in discipline construction. They can also fully mobilize the enthusiasm and creativity of professional and technical personnel through a sound talent evaluation and incentive mechanism. For irregularities in the aging market, the relevant departments still need to strengthen the legalization of aging-related industries and regulate the market order, such as improving the social insurance law and establishing a universal long-term care insurance system, using laws and regulations to provide support for the benign development of the silver-haired industries.

In addition, the eastern region is developing ahead of the central and western regions, so it is urgent to learn from the eastern experience to promote the industrial upgrading driving effect of the aging-related consumption trend in the central and western regions. Population aging is an irreversible trend; the central and western regions should focus on fostering the vitality of the silver-haired market while developing locally advantageous industries. Local governments should target the development of aging services and focus on the training of professionals, tax breaks, and subsidies for home purchase and residence are needed to enhance the willingness of professional talents to reside locally, following the example of the eastern region to accumulate capital for the development of the technical services through the retention of talents.

### Limitations and prospects

While this study provides valuable insights, it is important to acknowledge its limitations and propose future research directions. Firstly, this study fails to discuss the effect of different levels of aging because of data missing. Significant differences in cognition and habits between older and younger seniors can lead to specific consumption tendencies, which have uncertain effects on industrial structure adjustment. Further quantification of elderly population number in different age groups is needed to clarify their impact on industrial low-carbon development. Secondly, due to data limitations, some specific service industries closely related to aging demands, such as the long-term care industry, were not considered in this study. But from a long-term perspective, the health care industry is a crucial growth point for the future development of the silver-haired services. Therefore, expanding the dataset to identify synergies between the growth of the elderly population and the development of the long-term care industry and its contribution to low-carbon economy is an important direction for future research.

## Footnotes

1 Net energy heating value *G*_*j*_ data from Appendix 4 of the China Energy Statistics Yearbook, carbon emission factor *A*_*j*_ from IPCC, carbon oxidation factor *B*_*j*_ from the National Climate Change Coordination Group Working Group III.

2 Eastern Region: Beijing, Tianjin, Hebei, Liaoning, Shanghai, Jiangsu, Zhejiang, Fujian, Shandong, Guangdong, Hainan. Central region: Shanxi, Inner Mongolia, Jilin, Heilongjiang, Anhui, Jiangxi, Henan, Hubei, Hunan. Western region: Guangxi, Chongqing, Sichuan, Guizhou, Yunnan, Shaanxi, Gansu, Qinghai, Ningxia, Xinjiang.
